# Development and verification of the PAM50-based Prosigna breast cancer gene signature assay

**DOI:** 10.1186/s12920-015-0129-6

**Published:** 2015-08-22

**Authors:** Brett Wallden, James Storhoff, Torsten Nielsen, Naeem Dowidar, Carl Schaper, Sean Ferree, Shuzhen Liu, Samuel Leung, Gary Geiss, Jacqueline Snider, Tammi Vickery, Sherri R. Davies, Elaine R. Mardis, Michael Gnant, Ivana Sestak, Matthew J. Ellis, Charles M. Perou, Philip S. Bernard, Joel S. Parker

**Affiliations:** NanoString Technologies, Inc, 530 Fairview Avenue North, Suite 2000, Seattle, WA 98109 USA; Genetic Pathology Evaluation Centre, Vancouver Coastal Health Research Institute and British Columbia Cancer Agency, 2655 Oak St, Vancouver, BC V5Z 1M9 Canada; Statistical consultant, New York, NY USA; Washington University School of Medicine, 660 S Euclid, St. Louis, MO 63110 USA; Department of Surgery and Comprehensive Cancer Center, Medical University of Vienna, Vienna, Austria; Centre for Cancer Prevention, Wolfson Institute of Preventive Medicine, Queen Mary University of London, Charterhouse Sq, London, EC1M 6BQ UK; Lester and Sue Smith Breast Center, Baylor College of Medicine, One Baylor Plaza, MS 600, Houston, TX 77030 USA; Lineberger Comprehensive Cancer Center, Department of Genetics, University of North Carolina at Chapel Hill, 450 West Drive, Chapel Hill, NC 27599 USA; Huntsman Comprehensive Cancer Center, Department of Pathology, 2000 Circle of Hope, Salt Lake City, UT 84103 USA

## Abstract

**Background:**

The four intrinsic subtypes of breast cancer, defined by differential expression of 50 genes (PAM50), have been shown to be predictive of risk of recurrence and benefit of hormonal therapy and chemotherapy. Here we describe the development of Prosigna™, a PAM50-based subtype classifier and risk model on the NanoString nCounter Dx Analysis System intended for decentralized testing in clinical laboratories.

**Methods:**

514 formalin-fixed, paraffin-embedded (FFPE) breast cancer patient samples were used to train prototypical centroids for each of the intrinsic subtypes of breast cancer on the NanoString platform. Hierarchical cluster analysis of gene expression data was used to identify the prototypical centroids defined in previous PAM50 algorithm training exercises. 304 FFPE patient samples from a well annotated clinical cohort in the absence of adjuvant systemic therapy were then used to train a subtype-based risk model (i.e. Prosigna ROR score). 232 samples from a tamoxifen-treated patient cohort were used to verify the prognostic accuracy of the algorithm prior to initiating clinical validation studies.

**Results:**

The gene expression profiles of each of the four Prosigna subtype centroids were consistent with those previously published using the PCR-based PAM50 method. Similar to previously published classifiers, tumor samples classified as Luminal A by Prosigna had the best prognosis compared to samples classified as one of the three higher-risk tumor subtypes. The Prosigna Risk of Recurrence (ROR) score model was verified to be significantly associated with prognosis as a continuous variable and to add significant information over both commonly available IHC markers and Adjuvant! Online.

**Conclusions:**

The results from the training and verification data sets show that the FDA-cleared and CE marked Prosigna test provides an accurate estimate of the risk of distant recurrence in hormone receptor positive breast cancer and is also capable of identifying a tumor's intrinsic subtype that is consistent with the previously published PCR-based PAM50 assay. Subsequent analytical and clinical validation studies confirm the clinical accuracy and technical precision of the Prosigna PAM50 assay in a decentralized setting.

**Electronic supplementary material:**

The online version of this article (doi:10.1186/s12920-015-0129-6) contains supplementary material, which is available to authorized users.

## Background

A significant body of evidence gathered over the course of more than 10 years has repeatedly demonstrated the prognostic significance and predictive ability of the four intrinsic subtypes of breast cancer (Luminal A, Luminal B, HER2-enriched, and Basal-like) [1–8], which were first described in 2000 by Perou *et al.* [9]. These studies began with genome-wide gene expression profiling from microarray datasets and progressed to a PCR-based test with a curated list of 50 genes (the “PAM50” gene signature) to classify breast tumors into one of these four subtypes [10]. Recently, the NanoString nCounter Dx Analysis System has been shown to provide more precise and accurate measures of mRNA expression levels in formalin-fixed, paraffin-embedded (FFPE) tissue when compared to PCR [11]. Polymerase-based assays require excessive optimization from FFPE tissues and can introduce biases in amplification as mRNA from FFPE tissue is highly fragmented and cross-links to protein during fixation. The NanoString nCounter Dx Analysis System provides a digital profile of up to 800 genes in a single hybridization reaction using no enzymes and a simple workflow [12]. Several research groups have recently transitioned from profiling oncology biomarkers in fresh frozen and FFPE tissue using enzyme chemistry-based expression analysis platforms to profiling FFPE tissue on the nCounter, while maintaining the clinical accuracy of their signatures [13–16].

Rather than simply implementing the existing PCR-based PAM50 signature on the NanoString platform, scientific best practices dictate that a *de novo* retraining of the PAM50 breast cancer intrinsic subtype classifier should be performed on the nCounter in order to develop the most accurate and robust classifier. The primary aim of this study was to train a PAM50-based subtype classifier and prognostic risk of recurrence (ROR) model on the NanoString nCounter Dx Analysis System that is consistent with the published qRT-PCR-based PAM50 assay using FFPE breast cancer tissue samples obtained specifically for this training. The second aim was to verify that the clinical accuracy of the NanoString Prosigna algorithm is equivalent to the PCR-based classifier and ROR model [10] using a test set of FFPE breast cancer samples independent of the training set.

## Methods

### Feasibility: cross platform evaluation

Feasibility experiments were conducted to test the concordance between gene expression measured on the NanoString nCounter and qRT-PCR. NanoString probes were designed to match the 50 classifier genes and 5 housekeeper genes defined by Parker *et al.* [10]. The feasibility experiments on the NanoString nCounter were carried out using NanoString’s standard life sciences custom CodeSets, consumables, and assay procedures [12]. The PCR-based PAM50 assays have been previously described [10].

Reproducibility of the NanoString versus qRT-PCR gene expression measurements was assessed for all 50 target genes and 5 housekeeping genes using 113 samples from archived formalin-fixed tumor blocks that were collected under an Institutional Review Board-approved protocol from Washington University. Cores from each tissue block were obtained using a disposable sampling tool (Harris Unicore, 1.2 mm) and total RNA was isolated using the Roche High-Pure RNA Paraffin kit (Roche Applied Science, Indianapolis, IN) [10]. RNA input for the FFPE samples ranged from 250 ng (nCounter) to 1.0 μg for qRT-PCR assays. High quality RNA isolated from matched fresh-frozen blocks (previously described in Parker *et al.* [10]) was also analyzed (at 100 ng per NanoString reaction) from a subset of the cases to assess the repeatability of the assay across the same specimen with different post-processing handling procedures. The intraclass correlation (ICC) was used to assess reproducibility by sample [17]. Accuracy of gene expression estimates were determined relative to qRT-PCR for a subset of 71 of the 113 FFPE samples. Additionally, accuracy of both platforms was examined using the published PCR-based classifier [10].

### Training clinical specimen collection

The criteria used to select samples for training included tissue amount and quality, along with each FFPE tissue block representing a unique patient. The complete inclusion criteria for algorithm training are described in Additional file 1: Table S1. Prior clinical data were not used to pre-select individual samples within each cohort used for subtype training. Four sets of FFPE breast cancer patient samples were used for training the Prosigna Algorithm (see Additional file 2: Table S2 for additional details). A first set of breast tumor tissue specimens (“BC no AST”) [18] with clinical outcome data was collected at the Genetic Pathology Evaluation Centre (University of British Columbia) and used for both Prosigna ROR score and subtype centroid training. These patients received no adjuvant systemic therapy (no AST), based on provincial guidelines at that time for clinically low risk women. A second set of breast tumor tissue specimens for subtype centroid training was collected at the University of North Carolina (UNC) from patients seen at UNC hospital [10]. A third set of breast tumors for subtype training was collected at Washington University in St. Louis (Wash U) [10, 19] from patients with invasive breast cancer after surgical excision. The fourth set of patient specimens were 24 FFPE reduction mammoplasty samples obtained from the Genetic Pathology Evaluation Centre.

The UNC and Wash U samples were included to ensure a broad demographic representation (e.g. approximately 30 % and 40 % African American women respectively) as part of the prototypical subtype centroid training exercise. The 24 FFPE reduction mammoplasty samples were included to ensure the training exercise recapitulated the experimental design of the training for the published PCR-based PAM50 assay [10], which also contained reduction mammoplasty samples as part of centroid training.

An independent set of FFPE breast cancer patient samples (BC TAM) with clinical outcome data were used for verifying the prognostic value of the trained Prosigna ROR score and subtype centroids. These specimens were also collected from the Genetic Pathology Evaluation Centre from estrogen receptor-positive (ER+), node-negative patients treated with five years of adjuvant tamoxifen [4]. A subset of the BC TAM samples consisted of RNA previously isolated from FFPE tissue, as part of Nielsen *et al.* [4]*.* These samples were included in the algorithm verification to increase the number of samples from the BC TAM study as not all patient blocks contained sufficient tissue for re-isolation.

### Prosigna tissue review, shipping and storage

Prior to performing a Prosigna assay, a certified pathologist reviewed all FFPE tissue blocks to identify and circle the area of viable invasive carcinoma. Two 1.0 mm diameter tissue cores were taken from the identified area of the block and shipped from the collection location to NanoString. For a small subset of the training samples, only one core was taken due to limited tumor within the identified area of invasive carcinoma. For the normal tissue samples, the certified pathologist reviewed the FFPE block to confirm the absence of any tumor tissue. Since the normal tissue samples contain a heterogeneous mixture of stromal and epithelial tissue, normal samples were assayed as FFPE tissue scrolls (10 um thickness) rather than core punches.

### NanoString Prosigna assay

RNA was isolated using a Roche column-based RNA extraction kit manufactured to NanoString’s specifications [20]. Briefly, paraffin was removed with D-limonene and tissue cores were digested overnight with proteinase-k. Digested samples were bound to a silica column, followed by an on-column DNase treatment for the removal of genomic DNA. Isolated RNA was eluted in a 30 μL volume and tested using a spectrophotometer to ensure it met the specifications for concentration (≥12.5 ng/μL) and purity (OD 260/280 nm 1.7-2.5).

The RNA was analyzed on the NanoString nCounter Dx Analysis System which delivers direct, multiplexed measurements of gene expression through digital readouts of the abundance of mRNA transcripts [12, 20]. The nCounter Dx Analysis System uses gene-specific probe pairs that hybridize directly to the mRNA sample in solution eliminating any enzymatic reactions that might introduce bias in the results. A Reporter Probe carries the fluorescent signal; a Capture Probe allows the complex to be immobilized for data collection. The Prosigna assay simultaneously measures the expression levels of 50 target genes [10] plus eight endogenous control (housekeeping) genes [10, 21, 22] in a single hybridization reaction using an nCounter CodeSet. Each assay also includes six positive quality controls comprised of a linear titration of *in vitro* transcribed RNA transcripts and corresponding probes, and eight negative quality controls consisting of probes with no sequence homology to human RNA sequences [23]. Each Prosigna assay run includes a reference sample consisting of *in vitro* transcribed RNAs of the 58 targets that are used for normalization purposes.

Sample processing steps after hybridization are automated on the nCounter Prep Station. The excess probes are removed followed by binding of the probe-target complexes on the surface of the nCounter cartridge via a streptavidin-biotin linkage. Probe-target complexes are aligned and immobilized in the nCounter cartridge. After sample processing has completed, cartridges are placed in the nCounter Digital Analyzer for data collection. Each target molecule of interest is identified by the target specific “color code” generated by six ordered fluorescent spots present on the reporter probe. The Reporter Probes on the surface of the cartridge are then counted and tabulated for each target molecule.

### Analysis methods

Hierarchical clustering was performed using Pearson’s correlation as the distance metric and average linkage clustering. The R package SigClust was used to assess significance of each cluster in order to identify the prototypical samples that were used to derive the centroids. Prototypical subtype centroids were assigned based on gene expression profiles concordant to previously published data [2, 3, 10] and subtype specific characteristics described by Carey and Perou [24]. The accuracy of the subtype assignments was assessed based on the following two criteria:

1. a Luminal A centroid that classifies tumors with the best prognosis as Luminal A relative to other putative Luminal A centroids
2. similar hazard ratios between Luminal A subtype and other high risk subtypes compared to previously published data

A multivariable Cox model was fit using ridge regression [25] in the R package glmnet to learn the ROR coefficients. The endpoint for risk of recurrence calculations was recurrence-free survival (RFS), defined as the interval from diagnosis until local, regional or distant recurrence or death due to breast cancer. Contralateral breast cancer and death due to causes other than breast cancer were treated as censoring events. Death due to breast cancer where a recurrence was not recorded was treated as an event with the event date being the date of death.

Using the BC TAM patient samples and following procedures outlined in Parker *et al.* [10], the C-index was used to assess the prognostic accuracy of the ROR model [26]. Briefly, from a given patient population, the C-index is calculated by first comparing ROR scores in all pairs of subjects in the population. The ROR values ranked for each pair are then compared to the differences in the rank ordered survival time to see if ROR was accurately ordering the outcome of each pair. A C-index of 0.5 indicates a classifier that does not estimate outcome better than random choice. A higher C-Index (up to a value of 1) indicates a model that more accurately estimates the true risk of recurrence.

## Results

### Feasibility: Cross platform evaluation

The median block age of 113 FFPE tumor samples used to compare NanoString and qRT-PCR repeatability was 10 years with a range of 7–13 years old. The gene expression of the 50 classifier genes for each sample was independently normalized to the geometric mean of five housekeeper genes. The median coefficient of variation was used to summarize the reproducibility of the expression measurement of each gene (Additional file 3: Figure S1A). These values ranged from 1.7 % to 6.7 % with a mean of 3.6 %, which were similar to those observed in the qRT-PCR replicates, and were not correlated with sample block age. The intraclass correlation (ICC) was used to assess reproducibility by sample [17]. Intraclass correlation values ranged from 0.964 to 0.999 with a mean of 0.993 (Additional file 3: Figure S1B). The highly reproducible expression estimates resulted in 98 % concordant subtype assignments and the ICC of the risk of relapse score was 0.998 (Additional file 3: Figure S1C). Similar results were demonstrated in all cases for fresh frozen samples (not shown).

nCounter gene expression estimates were concordant to qRT-PCR with a median ICC of 0.90 (mean = 0.85) despite a slight decrease in sensitivity evident by a mean slope between nCounter and qRT-PCR data of 0.88 across the 50 classifier genes (Additional file 4: Table S3). As expected, those genes that exhibited the lowest ICC and the lowest slopes were also the least differentially expressed across all patients in this cohort. Accuracy estimates for all genes are shown in Additional file 4: Table S3.

The published PCR-based classifier also generated accurate calls by the NanoString nCounter Dx Analysis System when compared to calls by PCR with 94 % concordance in subtype calls and ICC values of 0.98 and 0.95 for the ROR-S and proliferation measures, respectively (Additional file 5: Figure S2). Given its technical and practical advantages and based on the high degree of concordance in these feasibility experiments, the NanoString nCounter was shown to be well suited to develop an *in vitro* diagnostic assay.

### Patient clinical characteristics for training and verification

**Fig. 1:**
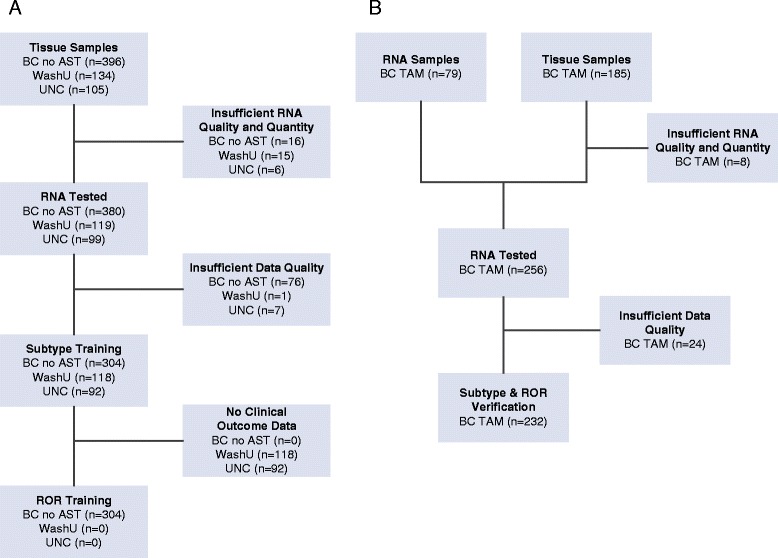
CONSORT diagram describing the breakdown for sample processing. Diagrams for (**a**) subtype and ROR training and (**b**) subtype and ROR verification

Eight hundred and twenty (820) patient FFPE breast tumor samples and 79 previously isolated RNA samples were received at NanoString and met the predefined sample inclusion criteria. After isolation of RNA from the tumor samples and assessment of yield and quality, 854 of these samples were analyzed on the NanoString nCounter Dx Analysis System. Gene expression data from 746 patient samples were determined to be of high quality and were used for algorithm training and verification (samples with low gene expression signals were excluded to minimize noise in algorithm training). A full description of clinical characteristics by cohort is included in Table 1 and the breakdown of sample processing is included in Fig. 1. Twenty four (24) FFPE breast reduction mammoplasty samples were received at NanoString and all yielded sufficient RNA with high quality gene expression data to be used in training the breast cancer normal samples centroids.

**Table 1:** Clinical characteristics by cohort for samples used for algorithm training

Characteristic	BC no AST	WashU	UNC	BC TAM
Median Follow Up (Years)	11.9	NA	NA	12.1
Patient Age				
Mean	59.9	59.3	56.5	66.3
Stdev	13.8	15.2	15.9	9.6
Premenopausal				
Yes	81	0	0	10
No	214	0	0	219
Unknown	9	118	92	3
ER Status				
Positive	203	65	47	232
Negative	99	48	30	0
Unknown	2	5	15	0
Node Status				
Positive	18	57	41	0
Negative	276	58	35	232
Unknown	10	3	16	0
HER2 Status				
Positive	44	31	21	19
Negative	253	59	51	210
Unknown	7	28	20	3
PR Status				
Positive	138	50	34	130
Negative	139	63	38	86
Unknown	27	5	20	16
Tumor Size				
≤2	193	87	24	149
>2	111	29	49	83
Unknown	0	2	19	0
Grade				
1	26	5	10	15
2	127	32	20	103
3	145	78	43	108
Unknown	6	3	19	6

Any missing values were not available or not collected and therefore not reportable

### Prototypical tumor centroid training

**Fig. 2:**
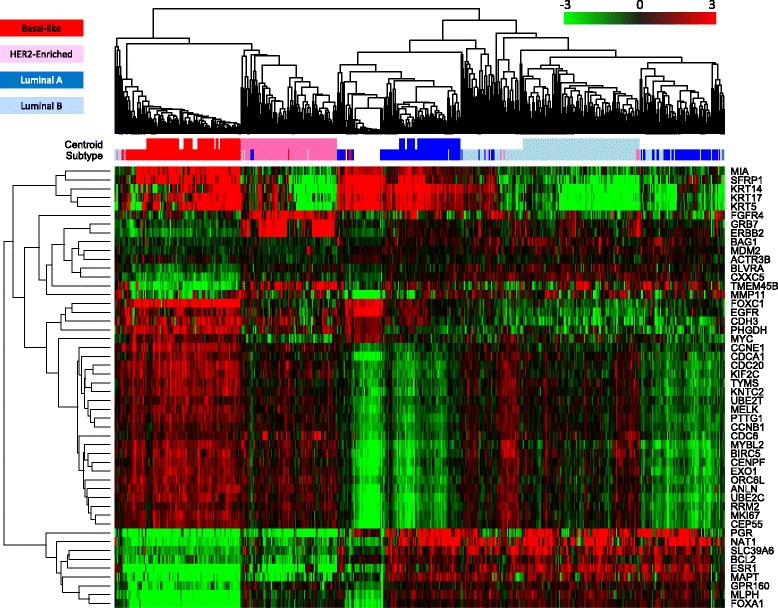
Hierarchical clustering of all subtype training samples. Clustering analysis (using a Pearson’s distance metric and average linkage) was performed on the median centered normalized, Log2 transformed data. The centroid color bars below the sample dendrogram represent the significant clusters that were chosen to establish each tumor centroid. The subtype color bars represent the subtype calls using the final algorithm. Since the reduction mammoplasty normal tissue samples do not contain tumor, they were not assigned a subtype and are represented as blanks in the subtype color bars

The four prototypical tumor subtype centroids (Basal-Like, HER2-Enriched, Luminal A, and Luminal B) were defined by identifying statistically significant (P < 0.001) clusters from hierarchical clustering of the PAM50 genes in 514 samples from the BC no AST, UNC, and WashU cohorts and 24 reduction mammoplasty samples (Fig. 2).

The gene expression in the prototypical clusters for each of the four tumor subtypes were similar to previously published results [2, 3, 10, 24, 27]. Examples of some of the markers used to identify each prototype include a Basal-Like cluster with highly expressed cytokeratin (KRT 5, 14, 17) and cell proliferation genes and low expression in genes associated with estrogen responsiveness and *ERBB2*. The HER2-Enriched cluster showed low expression of estrogen-related genes; whereas cell proliferation genes were highly expressed as well as *ERBB2*, *GRB7*, and *FGFR4*. Genes associated with estrogen responsiveness were elevated in the both Luminal A and Luminal B clusters though the Luminal B cluster had much higher expression in cell proliferation genes than the Luminal A cluster. Patient samples from each of three training cohorts were represented in each of the four prototypical groups/centroids (Additional file 6: Table S4). None of the reduction mammoplasty samples were contained within any of the significant tumor subtype clusters used to define the centroids. Principal component analysis was performed on the gene expression data from the training cohorts to determine the primary source of variability in these patient samples. The first three out of fifty principal components separate the samples based on intrinsic tumor biology and are the major source (63 %) of variability in the gene expression data in the training samples. In contrast, the cohort (or institution the sample was collected at) was not a major source of variation in the principle component analysis (Additional file 7: Figure S3).

**Fig. 3:**
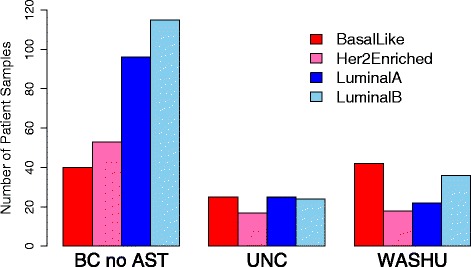
Distribution of subtypes from subtype training samples. Log2 transformed, reference sample and geomean normalized, and gene scaled nCounter data from the BC no AST, WashU, UNC cohorts assessed by the trained NanoString Prosigna algorithm

In order to standardize the distribution of gene expression across all 50 classifier genes, a Z-score transformation (calculated from the training samples) was applied to the normalized data. The transformed data were then used to set 50 gene centroids for each of four tumor subtypes. Subtypes were subsequently assigned for all 514 (BC no AST, WashU, and UNC) tumor samples based on the maximum Pearson’s correlation between the transformed gene expression for each sample and the four tumor centroids (Additional file 8: Figure S4). The distribution of subtypes from the BC no AST, WashU, UNC cohorts are illustrated in Fig. 3.

**Fig. 4:**
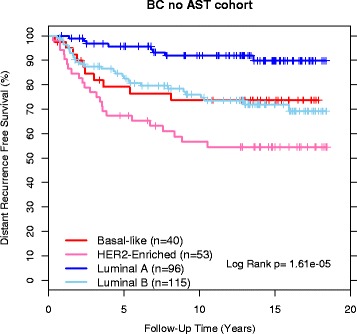
DRFS Kaplan–Meier plot for subtypes for ROR training cohort. Subtype colors and numbers of patients are included in the plot along with the results from the Log Rank test

The accuracy of the Prosigna subtype assignments was assessed by examining the outcomes of the untreated patients from the BC no AST cohort. Compared to alternate approaches, patients assigned a Luminal A subtype with the Prosigna centroids and Pearson’s distance metric had the best prognosis (assessed by Kaplan-Meier survival curves of the BC no AST cohort) compared to the other three subtypes (Fig. 4) as observed previously for the PCR-based PAM50 classifier [10]. Outcomes for the higher risk subtype tumors in this no AST cohort also mirror what was seen in a previous PAM50 analysis with the basal and Her2-enriched subtype tumors showing a higher risk of early recurrence while the luminal B tumors have a chronic risk of recurrence. This chronic risk of recurrence is evident in Fig. 4 where by 10 years the luminal B patients have a similarly high risk of recurrence compared to the basal-like patients.

Subtype classifications from BC no AST patient samples were also compared to subtype classifications from an untreated cohort of the Netherlands Cancer Institute (NKI) [28]. Comparisons with NKI were carried out to verify that the NanoString subtype classifier was predictive of 10 year recurrence-free survival with hazard ratios similar to those observed in previous PCR-based PAM50 training exercises [10]. The NKI gene expression data were generated on microarrays and a platform correction was necessary for these comparisons. The subtype assignments were fit using a Cox proportional hazard model to estimate the hazard ratio of each subtype. This was performed separately for each cohort (BC no AST and NKI) using both the published PCR-based predictor and the Prosigna predictor. This analysis was carried out using 10 year recurrence free survival (RFS) as the endpoint. Table 2 illustrates that the calculated relative risk of the three higher risk subtypes (relative to Luminal A) is similar between the two classifiers, even when the data were collected on two different platforms. As outlined above, in British Columbia at the time of collection, provincial guidelines called for no adjuvant systemic therapy (no AST) for clinically low risk patients. In contrast, the population in the NKI cohort was quite heterogeneous and included a significant number of patients with high risk clinical characteristics (pre-menopausal, node-positive, and large tumors). Due to this variation in clinical risk factors, the BC no AST and the NKI patient populations showed significant differences in absolute risk (Additional file 9: Figure S5); however, despite these differences, the Prosigna predictor provides similar proportional hazard estimates to the published predictor in both cohorts.

**Table 2:** Proportional hazard ratios for N0 patients in BC no AST and NKI cohorts

Cohort	NKI		BC no AST	
Predictor	PCR-based	Prosigna	PCR-based	Prosigna
Luminal B	4.19	3.37	3.47	3.18
HER2-enriched	5.19	4.88	4.78	5.02
Basal-like	2.41	2.45	3.17	2.96

Results generated using the published classifier and the Prosigna classifier

### ROR Score Training

**Fig. 5:**
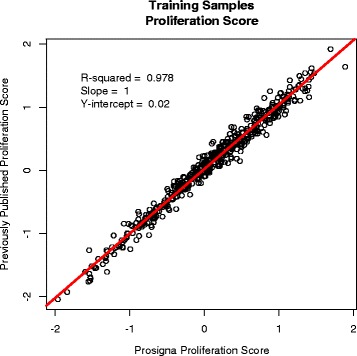
Plotted pairs of the Prosigna proliferation score and the previously published [4] proliferation score. Individual points are from the algorithm training samples (*n* = 514). The R-squared, slope, and Y-intercept of the comparison are shown in the top left of the plot

A 50-gene ROR score model and simplified 46-gene ROR model (removing BIRC5, CCNB1, GRB7, and MYBL2) were developed using multivariable Cox modeling with ridge regression to learn the ROR score coefficients. These 4 genes were removed and the 46 gene model compared to the 50 gene model as they did not seem to add prognostic accuracy to the predicted risk of recurrence. The ROR score incorporate the biology of the intrinsic subtypes by including the Pearson’s coefficients to the four tumor centroids as factors in the model in addition to a proliferation score and primary tumor size as additive terms to predict risk of recurrence. A number of PAM50 based ROR models have been previously reported [4, 10] which incorporate different variables including a subtype only model (ROR-S), a subtype and tumor size model (ROR-T or ROR-C), and a subtype and a proliferation score model (ROR-P). The variables included in the Prosigna ROR model are consistent with those included in the PCR-based ROR-PT model first reported by Nielsen *et al.* [4]. The proliferation score was calculated using the arithmetic mean (average) of the normalized and transformed expression of a subset of the 50 classifier genes (Additional file 10: Table S5) that are associated with cell cycle progression and which were shown to be co-regulated in the gene-associated dendrogram from the training set hierarchical cluster (Fig. 2). The Prosigna proliferation score is highly correlated to the published proliferation score described by Nielsen *et al.* [4] (Fig. 5).

The resulting Prosigna ROR score is calculated using weighted coefficients to the four subtypes, a proliferation score, and tumor size. Tumor size was calculated by assigning a binary classification value to primary tumors measuring 2.0 cm or less in the greatest dimension versus those measuring greater than 2.0 cm. The calculated Prosigna ROR scores were adjusted to a 0–100 scale. Scaling factors were generated using the entire range of Prosigna ROR scores from all tumor samples from algorithm training and verification (UNC, WashU, BC no AST, and BC TAM) where tumor sizes were available.

**Fig. 6:**
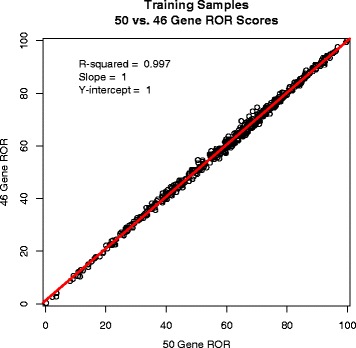
Plotted pairs of 46 gene and 50 gene ROR values. Individual points are from 514 algorithm subtype training samples. The R-squared, slope, and Y-intercept of the comparison are shown in the top left of the plot

The similarity of the 46 gene ROR and 50 gene ROR scores was evaluated. Data were generated from all the training samples (UNC, WashU, and BC no AST) using both models. When the paired data for each model were plotted against each other the removal of 4 genes had an insignificant impact on the reported ROR score with an R-squared value equal to 0.997 (Fig. 6) indicating they are functionally the same score.

### Verification of prototypical centroids and Prosigna ROR score

**Fig. 7:**
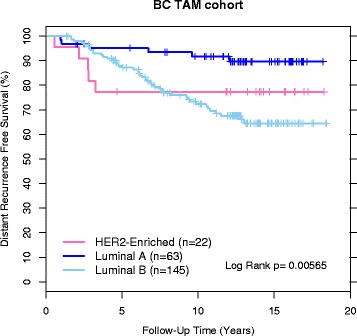
DRFS Kaplan–Meier plot for subtypes for the ROR verification cohort. Subtype colors and numbers of patients are included in the plot along with the results from the Log Rank test

The accuracy of the Prosigna classifier was verified in the independent BC TAM cohort by comparing the outcomes of the patients with a Luminal A subtype with the other subtypes. Similar to what was reported for the published PCR-based PAM50 classifier [4], patients assigned a Luminal A subtype had superior outcome (assessed by Kaplan-Meier survival curves of the BC TAM cohort) compared to the other two subtypes (Fig. 7) with similar calculated hazard ratios (Luminal B 3.87 and Her2 Enriched 2.86 versus Luminal B 3.67 and Her2 Enriched 2.80 for published versus Prosigna respectively). Two out of the 232 BC TAM patient samples were classified as Basal-Like and these were excluded from the Kaplan-Meier analysis due to insufficient sample size to make any meaningful conclusions.

**Fig. 8:**
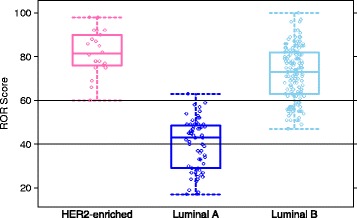
Boxplots showing the distribution of ROR scores for N0 BC TAM patient tumor sample. Results were grouped based on tumor classification as one of three breast cancer subtypes. The limits of the boxes represent the first and third quartile and the whiskers represent +/−1.58 IQR/sqrt(n). The horizontal dashed lines illustrate the ROR cutoffs for low/intermediate and intermediate/high risk for N0 patients. Individual data points are jittered for illustration purposes

The distribution of the ROR scores for BC TAM patient tumors from each of these three subtypes show that the model appropriately characterizes the risk associated with PAM50 intrinsic subtype. Only Luminal A tumors were classified as low risk with ROR scores less than 40. Other than a single Luminal A tumor with an ROR score of 63, only Luminal B or HER2-enriched tumors were classified as high risk with ROR scores greater than 60 (Fig. 8). The distribution for these three subtypes was confirmed in the two subsequent validation studies [29, 30] where in over 2000 patient samples Luminal A subtype tumors represented less than 0.3 % of samples with ROR > 60 and were the only subtype with ROR < 40 (Additional file 11: Figure S6). Nielsen *et al*. [4] describes the BC TAM cohort as biased towards higher risk breast cancers due to Provincial treatment guidelines in place at the time when these patient samples were collected, which consequently biases the cohort toward higher risk subtypes and higher average ROR scores. This cohort bias is further illustrated in the overall risk of the BC TAM patients who received tamoxifen treatment having outcomes similar to the BC no AST cohort (described herein as low risk) who received no adjuvant systemic therapy (Additional file 9: Figure S5). In the subsequent validation studies of Prosigna, which are likely more representative of the general ER+ patient population than the BC TAM cohort, a broader distribution of ROR scores was observed, including many more patients with very low ROR scores (<20).

**Fig. 9:**
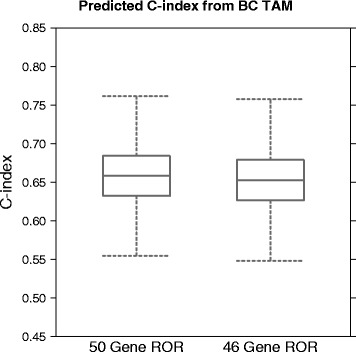
C-index of 46 and 50-gene ROR scores for distant recurrence-free survival. The limits of the boxes represent the first and third quartile and the whiskers represent +/−1.58 IQR/sqrt(n)

The accuracy of the 46 and 50-gene ROR models were then assessed. Predictions of the C-index for each model were generated from 1,000 bootstrap samples of the risk assignments from the BC TAM verification data using the R package Hmisc. There was no difference in accuracy between the 46 or 50-gene ROR scores (Fig. 9). The fact that there is no clinical (Fig. 9) or functional (Fig. 6) difference between the two ROR models verifies that the four genes do not add prognostic accuracy to the predicted risk of recurrence. As the two models are equivalent, the 46 gene ROR model was chosen for NanoString’s PAM50-based Prosigna assay and subsequently validated in two previously published clinical studies [29, 30].

**Fig. 10:**
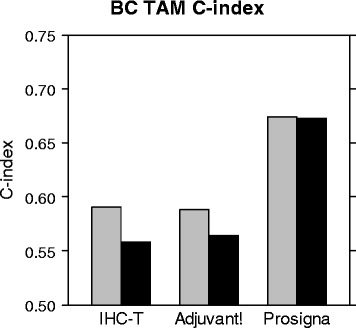
Accuracy of the Prosigna ROR score to predict DSS and DRFS compared to other models. Different histogram colors represent whether DSS (black) or DRFS (gray) was used as the clinical endpoint to test each model

Consistent with analysis performed by Nielsen *et al.* [4], the accuracy of the final continuous Prosigna ROR score model was verified using the Prosigna assay on node negative patients from the BC TAM series. Briefly, C-index was used to compare the accuracy of the Prosigna model to a model based on clinical variables (Adjuvant! Online), and a Ki67 and HER2 IHC based model that includes tumor size (IHC-T). BC TAM patients were all confirmed as ER+ by dextran-charcoal–coated ligand-binding assay so ER was not included in the IHC-T model. All models were evaluated for accuracy using distant recurrence-free survival (DRFS) as well as disease specific survival (DSS) as an alternate clinical endpoint (Fig. 10). The Prosigna model showed significant improvement over both IHC and Adjuvant! Online when either DRFS or DSS were used as the clinical endpoint. The Prosigna ROR score was determined to be a robust estimate of risk relative to the other models tested similar to what was previously reported in the published PCR-based PAM50 assay [4].

## Discussion

The four breast cancer intrinsic subtypes were first described by Perou *et al.* [9] and originally defined by differential gene expression of 1,753 genes. Subsequent studies confirmed the existence of these subtypes and further demonstrated that they were predictive of overall and relapse-free survival [1, 2]. The gene list was reduced to the 50 classifier genes in the PAM50 assay, while maintaining the biological classification and prognostic accuracy inherent to the intrinsic subtype prediction [10, 31]. The PCR-based PAM50 subtype classifier and ROR score are prognostic in estrogen receptor positive, post-menopausal women treated with endocrine therapy alone [4] and prognostic and predictive of hormonal therapy benefit in pre-menopausal women treated with adjuvant hormonal therapy [5]. PAM50 subtypes and PAM50 proliferation have also been shown to be predictive of benefit of chemotherapy [6, 7, 32] and of pathologic complete response in neoadjuvant chemotherapy studies [8, 10]. Additionally, these studies have shown that PAM50 provides a better estimate of prognosis and of prediction of treatment benefit than IHC-based surrogates [4–6].

Breast cancer subtypes, as defined using available IHC assessments of ER, PR, and HER2 biomarkers, have been included in treatment guidelines for breast cancer patients as a surrogate for molecular subtyping using PAM50 [33]. These biomarkers are critical in the initial diagnosis of breast cancer and for determining whether endocrine therapy or HER2-targeted therapy is necessary; however, studies have shown that subtypes derived from these 3 biomarkers are sub-optimal surrogates for the PAM50-based breast cancer intrinsic subtypes [5, 7, 34]. The addition of a fourth marker for cell proliferation (Ki-67) still does not accurately characterize the intrinsic biology needed to classify tumors into one of the four PAM50 subtypes. A combination of these four IHC markers can achieve approximately 70-80 % sensitivity and specificity when compared to gene expression classification [18]. Even a comparison of proliferation assessed by IHC-based Ki-67 was less accurate with respect to clinical outcome when compared to proliferation assessed by the RNA-based PAM50 proliferation score [6]. Additionally, recent studies have demonstrated that inter-laboratory reproducibility of IHC measurement of Ki-67 is insufficient for routine clinical practice [35]. Efforts are ongoing to improve the performance of IHC-based biomarker testing by standardizing quality control, training, methods and cutoffs, particularly with ER [36], Her2 [37] and Ki-67 [38].

Currently, the laboratory-developed BluePrint® test [39] is the only other marketed gene expression-based test that outputs breast cancer molecular subtypes. However, the 80 genes profiled in that assay were selected using fresh frozen tissue to classify tumors as Luminal-type, HER2-type, or Basal-type based on their concordance to ER, PR and HER2 IHC values. Additionally, the BluePrint test does not include genes that distinguish Luminal A and B intrinsic subtypes. The accuracy of this test when compared to the canonical gene expression-based intrinsic subtypes should be similar to subtyping by IHC which has demonstrated limitations [5, 7, 34].

There are several other gene expression based tests currently available that output a breast cancer risk classification [28, 40, 41]. The Endopredict score and Oncotype DX® Recurrence Score® were originally trained, tested and validated using FFPE derived RNA, where the MammaPrint® score was trained, tested and validated using RNA isolated from fresh frozen tissue. All three of these gene expression signatures were defined based on the ability to predict distant recurrence whereas the PAM50 genes were defined based on the ability to identify the underlying biology defined by the four intrinsic breast cancer subtypes, which are themselves predictive of distant recurrence. The Oncotype DX Recurrence Score is considered by some to be predictive of chemotherapy benefit [42]; however, some controversy exists regarding the bioinformatics approach used to make this claim [43] and the relevance of the chemotherapy regimens and patient population used within the clinical trials tested [44]. There are ongoing clinical trials to assess the clinical utility of the MammaPrint RS [45], Oncotype DX® Recurrence Score® [46] and Prosigna ROR [47] within the ER-positive, Her2-negative intended use population using modern chemotherapy regimens.

The feasibility experiments described herein showed highly concordant results between the NanoString nCounter platform and qRT-PCR. Across 113 FFPE breast cancer specimens, expression of the PAM50 genes, subtypes and ROR scores were highly concordant between qRT-PCR and nCounter using the PCR-based classifier. Additionally, in repeated measures from 71 FFPE tumor specimens, the published classifier on nCounter data gave highly concordant results for ROR and subtype. The similarity of results in the cross platform evaluation and within tissue repeatability was instrumental in the selection of the NanoString nCounter Dx Analysis System as the platform for development and training of the *in vitro* diagnostic version of the PAM50 classifier.

The subtype centroid and ROR model training for the Prosigna algorithm were designed to parallel the training of the published PCR-based PAM50 classifier and ROR model. This retraining was executed using an independent set of FFPE breast tissue samples, with RNA isolated using a GMP-manufactured kit, and using data solely collected on the NanoString nCounter Dx Analysis System. The gene expression profiles of the Prosigna intrinsic breast cancer subtype centroids are similar to the published PAM50 classifier centroids. Additionally, patients assigned a Luminal A subtype were significantly lower risk compared to the Luminal B, HER2-enriched, and Basal-like subtypes in the training population with no adjuvant systemic therapy as previously observed in the published PCR-based PAM50 classifier [10]. The results from this independent verification study further demonstrate that both Luminal B and HER2-enriched breast cancer are predictive of poorer distant recurrence free survival compared to Luminal A breast cancer in an ER+ node-negative tamoxifen-treated early-stage breast cancer population (a set containing very few basal-like subtype patients). The Prosigna ROR score training produced a model that predicts the risk of distant recurrence in the same ER+ node-negative tamoxifen-treated population. These results recapitulate the findings in the same patient population using the published PCR-based PAM50 classifier and ROR model [4] and provide additional evidence to the already dense landscape of published results showing that the four intrinsic subtypes defined by the PAM50 genes contain significant prognostic information.

The results from both the BC no AST patient population used for Prosigna training and the NKI data used to train the published PCR-based PAM50 classifier [10] show that the Luminal A and the low ROR patient populations have a low risk of recurrence for 10 years following surgery with no adjuvant therapy. This would suggest that there may be patients that are identified by the PAM50 genes with low enough risk to be spared adjuvant chemotherapy, or potentially even hormonal therapy. However, the lower risk profile of the Luminal A patients in the BC no AST population compared to the NKI population, suggests this gene expression-based information should be used in concert with other clinical covariates such as age, node status, and tumor size to most accurately identify such very low risk breast cancers. It should be noted that both of these data sets are derived from populations originally diagnosed in the 1980s or 1990s with relapse and survival rates that are poorer, overall, than for more contemporary sets of patients.

In contrast to the published PCR-based PAM50 classifier [10], which was developed to be a flexible research tool applicable across platforms and datasets, the Prosigna PAM50 assay is intended to be used as a clinical assay on a fixed platform with a fixed statistical model. One important necessary difference in the two algorithms is that Prosigna uses fixed values for Z-score transformation, while the PCR-based PAM50 algorithm should be platform and cohort adjusted for each new data set. Prosigna used the 212 UNC and WashU training samples to generate a static control as all four subtypes were well represented in this population with gene expression values generated on the same platform using the same clinical grade reagents, procedures, and the same normalization scheme. The Prosigna algorithm therefore requires no additional cohort or platform normalization, providing a stable and unchanging subtyping algorithm that can be applied to a single patient sample or to a biased patient population (such as all hormone receptor positive) with both accurate and stable results [29, 30]. In contrast, the PAM50 research version algorithm relies on each researcher to select an appropriate population and method to do the gene centering in order to normalize the population being tested back to the original training population of the published PCR-based PAM50 classifier [10]. Failing to do so correctly will result in biased subtype classifications that may be inconsistent with the clinicopathological features of the patient samples being tested [48]. However, for research studies using the PCR-based PAM50 classifier such as Nielsen *et al.* [4] where the normalization was performed correctly, the subtype distribution and associated clinical outcomes will be representative of each selected patient population and has been shown here to have very similar performance to Prosigna. Recently, the research version PAM50 centroid classification method has been criticized for these features, namely adjustment through normalization to each data set [49]; however, these criticisms are not germane to the Prosigna assay because it is all run on a single technology platform with a standardized technical normalization procedure, thereby providing a stable and robust subtyping assay that can be applied to samples at decentralized testing sites.

Of note, unlike the published classifier of Parker *et al.* [10], the Prosigna assay does not have a trained Normal-like centroid as a control to identify inclusion of contaminating non-tumor tissue. For the Prosigna assay a pathologist performs a tissue review on an H&E stained section to identify the area of viable invasive carcinoma on the FFPE breast tumor block which is appropriate for inclusion in to the assay. The assay procedure requires macrodissection to exclude adjacent non-tumor tissue from unstained slide-mounted sections using a matched H&E slide. The results from the subsequent analytical validation of Prosigna show that assay procedures and results are robust, with negligible impact on the assay of small-to-moderate amounts of remnant adjacent non-tumor tissue [50]. An interferents study performed by Elloumi *et al.* [51] tested the effect of RNA isolated from adjacent normal breast titrated into RNA from paired tumor. The PAM50 result with the published algorithm were systematically biased towards a lower ROR whereas research-based versions of MammaPrint and Oncotype Dx results from the same samples were generally biased towards lower risk, they also were sometimes biased towards higher risk scores. However, even when the titrated tumor RNA to adjacent normal RNA ratio was around 50 % only about half of the samples were called Normal-like [51]. This is likely due to the fact that the gene expression profiles of surrounding non-tumor tissue do not resemble distant uninvolved normal breast tissue [52, 53]. These observations further suggest a Normal-like control lacks the sensitivity and specificity required for inclusion in the Prosigna *in vitro* diagnostic test, but is suitable for identifying samples with substantial normal RNA contamination in a research setting where pathology review and macrodissection may not always be feasible.

The analytical [50] and clinical validation studies [29, 30] of the Prosigna assay were performed in an HR+ endocrine-treated early-stage breast cancer population using the previously defined and locked Prosigna algorithm derived from this training set on RNA derived from FFPE tissue. These studies demonstrated both analytical reproducibility and Level I evidence for clinical validity using archived specimens [54]. The analytical validation showed that the Prosigna ROR and subtype result was highly reproducible across multiple laboratory sites, users, and reagent lots supporting the decentralized use of the assay. The clinical studies showed Luminal B breast cancers are predictive of poorer DRFS compared to Luminal A breast cancer and the Prosigna ROR provides significant prognostic information over and above standard clinical variables. These results are consistent with the Prosigna verification study described herein. Results from the two clinical validation studies also show that the ROR score is predictive of risk of late distant recurrence after 5 years of hormonal therapy [55, 56].

## Conclusion

The Prosigna assay is the only genomic assay that is CE-marked and FDA-cleared that was trained, verified and validated to provide an accurate estimate of the risk of distant recurrence in hormone receptor positive breast cancer using RNA from FFPE breast cancer patient samples. Additionally, Prosigna is the only assay that is capable of classifying patients into one of the four breast cancer intrinsic subtypes using a classifier that was trained and verified to be consistent with the published PAM50 classifier.

## Additional files

Additional file 1: Table S1. Study inclusion and exclusion criteria for tumor subtyping. (DOCX 30 kb) Additional file 2: Table S2. Description of FFPE breast cancer patient samples used for training and verifying the Prosigna Algorithm. (DOCX 30 kb) Additional file 3: Figure S1. A) Coefficient of variation of each gene, B) interclass correlations for subtype call, and C) replicate ROR-S comparisons. Subtype and ROR-S were generated using the published PAM50 classifier, between NanoString nCounter replicate samples. (PDF 231 kb) Additional file 4: Table S3. Accuracy estimates (ICC, Slopes, and R-squared) for all genes between nCounter and qRT-PCR. (DOC 57 kb) Additional file 5: Figure S2. Comparison of ROR-S and proliferation between NanoString nCounter and PCR data. ROR-S and proliferation scores were generated using the published PCR-based PAM50 classifier. (PDF 101 kb) Additional file 6: Table S4. Number of patient samples from each of the three training cohorts. Numbers represent patient samples used to define each of the four tumor centroids. (DOCX 27 kb) Additional file 7: Figure S3. Principal component analysis performed on the gene expression data from the training cohorts. Data from each sample are colored by subtype classification (A) or cohort (B). (PDF 114 kb) Additional file 8: Figure S4. Four tumor subtype centroids of the Prosigna test. Data are Log2 transformed, reference sample and geomean normalized, and gene scaled nCounter data. (PDF 136 kb) Additional file 9: Figure S5. RFS Kaplan–Meier plot for BC No AST, BC TAM, and NKI cohorts. Cohort colors and numbers of patients are included in the plot along with the results from the Log Rank test. (PDF 59 kb) Additional file 10: Table S5. Genes used to calculate the Prosigna proliferation score. (DOC 28 kb) Additional file 11: Figure S6. Distribution of the ROR scores for ABCSG8 and transATAC patient tumor samples. The limits of the boxes represent the first and third quartile and the whiskers represent +/−1.58 IQR/sqrt(n) for each of the three subtypes shown. The horizontal dashed lines illustrate the ROR cutoffs for low/intermediate and intermediate/high risk for N0 patients. Individual points are jittered for illustration purposes. (PDF 83 kb)

## Abbreviations

ROR: Risk of recurrence
FDA: US Food and Drug Administration
FFPE: Formalin-fixed paraffin-embedded
PCR: Polymerase chain reaction
DRFS: Distant recurrence-free survival
DSS: Disease specific survival
ICC: Intraclass correlation

## References

[1] Sorlie T, Perou CM, Tibshirani R, Aas T, Geisler S, Johnsen H (2001). Gene expression patterns of breast carcinomas distinguish tumor subclasses with clinical implications. Proc Natl Acad Sci U S A.

[2] Sorlie T, Tibshirani R, Parker J, Hastie T, Marron JS, Nobel A (2003). Repeated observation of breast tumor subtypes in independent gene expression data sets. Proc Natl Acad Sci U S A.

[3] Hu Z, Fan C, Oh DS, Marron JS, He X, Qaqish BF (2006). The molecular portraits of breast tumors are conserved across microarray platforms. BMC Genomics..

[4] Nielsen TO, Parker JS, Leung S, Voduc D, Ebbert M, Vickery T (2010). A comparison of PAM50 intrinsic subtyping with immunohistochemistry and clinical prognostic factors in tamoxifen-treated estrogen receptor-positive breast cancer. Clin Cancer Res.

[5] Chia SK, Bramwell VH, Tu D, Shepherd LE, Jiang S, Vickery T (2012). A 50-gene intrinsic subtype classifier for prognosis and prediction of benefit from adjuvant tamoxifen. Clin Cancer Res.

[6] Martin M, Prat A, Rodriguez-Lescure A, Caballero R, Ebbert MT, Munarriz B (2013). PAM50 proliferation score as a predictor of weekly paclitaxel benefit in breast cancer. Breast Cancer Res Treat.

[7] Cheang MC, Voduc KD, Tu D, Jiang S, Leung S, Chia SK (2012). Responsiveness of intrinsic subtypes to adjuvant anthracycline substitution in the NCIC.CTG MA.5 randomized trial. Clin Cancer Res.

[8] Prat A, Bianchini G, Thomas M, Belousov A, Cheang MC, Koehler A (2014). Research-based PAM50 subtype predictor identifies higher responses and improved survival outcomes in HER2-positive breast cancer in the NOAH study. Clin Cancer Res.

[9] Perou CM, Sorlie T, Eisen MB, van de Rijn M, Jeffrey SS, Rees CA (2000). Molecular portraits of human breast tumours. Nature.

[10] Parker JS, Mullins M, Cheang MC, Leung S, Voduc D, Vickery T (2009). Supervised risk predictor of breast cancer based on intrinsic subtypes. J Clin Oncol.

[11] Reis PP, Waldron L, Goswami RS, Xu W, Xuan Y, Perez-Ordonez B (2011). mRNA transcript quantification in archival samples using multiplexed, color-coded probes. BMC Biotechnol..

[12] Geiss GK, Bumgarner RE, Birditt B, Dahl T, Dowidar N, Dunaway DL (2008). Direct multiplexed measurement of gene expression with color-coded probe pairs. Nat Biotechnol.

[13] Lohavanichbutr P, Mendez E, Holsinger FC, Rue TC, Zhang Y, Houck J (2013). A 13-gene signature prognostic of HPV-negative OSCC: discovery and external validation. Clin Cancer Res.

[14] Lee J, Sohn I, Do IG, Kim KM, Park SH, Park JO (2014). Nanostring-based multigene assay to predict recurrence for gastric cancer patients after surgery. PLoS One.

[15] King LY, Canasto-Chibuque C, Johnson KB, Yip S, Chen X, Kojima K, et al. A genomic and clinical prognostic index for hepatitis C-related early-stage cirrhosis that predicts clinical deterioration. Gut. 2014;64(8):1296-302 10.1136/gutjnl-2014-307862 PMC4336233 25143343

[16] Scott DW, Wright GW, Williams PM, Lih CJ, Walsh W, Jaffe ES (2014). Determining cell-of-origin subtypes of diffuse large B-cell lymphoma using gene expression in formalin-fixed paraffin-embedded tissue. Blood.

[17] Shrout PE, Fleiss JL (1979). Intraclass correlations: uses in assessing rater reliability. Psychol Bull.

[18] Cheang MC, Chia SK, Voduc D, Gao D, Leung S, Snider J (2009). Ki67 index, HER2 status, and prognosis of patients with luminal B breast cancer. J Natl Cancer Inst.

[19] Graveel CR, DeGroot JD, Su Y, Koeman J, Dykema K, Leung S (2009). Met induces diverse mammary carcinomas in mice and is associated with human basal breast cancer. Proc Natl Acad Sci U S A.

[20] NanoString Technologies Inc: Prosigna™ Breast Cancer Prognostic Gene Signature Assay [Package Insert]. Seattle, WA: NanoString Technologies, Inc; 2013-2015.

[21] Majidzadeh AK, Esmaeili R, Abdoli N (2011). TFRC and ACTB as the best reference genes to quantify Urokinase Plasminogen Activator in breast cancer. BMC Res Notes..

[22] Szabo A, Perou CM, Karaca M, Perreard L, Palais R, Quackenbush JF (2004). Statistical modeling for selecting housekeeper genes. Genome Biol.

[23] Baker SC, Bauer SR, Beyer RP, Brenton JD, Bromley B, Burrill J (2005). The External RNA Controls Consortium: a progress report. Nature Methods.

[24] Carey LA & Perou CM. Gene Arrays, Prognosis, and Therapeutic Interventions. In: Jay R. Harris et al. Diseases of the breast. 4th ed. Philadelphia, PA: Lippincott Williams & Wilkins; 2009. pp. 458-472

[25] Verweij PJ, Van Houwelingen HC (1994). Penalized likelihood in Cox regression. Stat Med.

[26] Harrell FE, Lee KL, Mark DB (1996). Multivariable prognostic models: issues in developing models, evaluating assumptions and adequacy, and measuring and reducing errors. Stat Med.

[27] Bastien RR, Rodriguez-Lescure A, Ebbert MT, Prat A, Munarriz B, Rowe L (2012). PAM50 breast cancer subtyping by RT-qPCR and concordance with standard clinical molecular markers. BMC Med Genomics..

[28] van de Vijver MJ, He YD, van't Veer LJ, Dai H, Hart AA, Voskuil DW (2002). A gene-expression signature as a predictor of survival in breast cancer. N Engl J Med.

[29] Dowsett M, Sestak I, Lopez-Knowles E, Sidhu K, Dunbier AK, Cowens JW (2013). Comparison of PAM50 risk of recurrence score with oncotype DX and IHC4 for predicting risk of distant recurrence after endocrine therapy. J Clin Oncol.

[30] Gnant M, Filipits M, Greil R, Stoeger H, Rudas M, Bago-Horvath Z (2014). Predicting distant recurrence in receptor-positive breast cancer patients with limited clinicopathological risk: using the PAM50 Risk of Recurrence score in 1478 postmenopausal patients of the ABCSG-8 trial treated with adjuvant endocrine therapy alone. Ann Oncol.

[31] Prat A, Parker JS, Fan C, Perou CM (2012). PAM50 assay and the three-gene model for identifying the major and clinically relevant molecular subtypes of breast cancer. Breast Cancer Res Treat.

[32] Jorgensen CL, Nielsen TO, Bjerre KD, Liu S, Wallden B, Balslev E (2014). PAM50 breast cancer intrinsic subtypes and effect of gemcitabine in advanced breast cancer patients. Acta Oncol.

[33] Goldhirsch A, Winer EP, Coates AS, Gelber RD, Piccart-Gebhart M, Thurlimann B (2013). Personalizing the treatment of women with early breast cancer: highlights of the St Gallen International Expert Consensus on the Primary Therapy of Early Breast Cancer 2013. Ann Oncol.

[34] Prat A, Adamo B, Cheang MC, Anders CK, Carey LA, Perou CM (2013). Molecular characterization of basal-like and non-basal-like triple-negative breast cancer. Oncologist.

[35] Polley MY, Leung SC, McShane LM, Gao D, Hugh JC, Mastropasqua MG (2013). An international Ki67 reproducibility study. J Natl Cancer Inst.

[36] Hammond ME, Hayes DF, Wolff AC, Mangu PB, Temin S (2010). American society of clinical oncology/college of american pathologists guideline recommendations for immunohistochemical testing of estrogen and progesterone receptors in breast cancer. J Oncol Pract.

[37] Wolff AC, Hammond ME, Hicks DG, Dowsett M, McShane LM, Allison KH (2013). Recommendations for human epidermal growth factor receptor 2 testing in breast cancer: American Society of Clinical Oncology/College of American Pathologists clinical practice guideline update. J Clin Oncol.

[38] Dowsett M, Nielsen TO, A'Hern R, Bartlett J, Coombes RC, Cuzick J (2011). Assessment of Ki67 in breast cancer: recommendations from the International Ki67 in Breast Cancer working group. J Natl Cancer Inst.

[39] Krijgsman O, Roepman P, Zwart W, Carroll JS, Tian S, de Snoo FA (2012). A diagnostic gene profile for molecular subtyping of breast cancer associated with treatment response. Breast Cancer Res Treat.

[40] Paik S, Shak S, Tang G, Kim C, Baker J, Cronin M (2004). A multigene assay to predict recurrence of tamoxifen-treated, node-negative breast cancer. N Engl J Med.

[41] Filipits M, Rudas M, Jakesz R, Dubsky P, Fitzal F, Singer CF (2011). A new molecular predictor of distant recurrence in ER-positive, HER2-negative breast cancer adds independent information to conventional clinical risk factors. Clin Cancer Res.

[42] Paik S, Tang G, Shak S, Kim C, Baker J, Kim W (2006). Gene expression and benefit of chemotherapy in women with node-negative, estrogen receptor-positive breast cancer. J Clin Oncol.

[43] Ioannidis JP (2007). Is molecular profiling ready for use in clinical decision making?. Oncologist.

[44] Schmidt M, Untch M (2014). Prediction of benefit from chemotherapy in ER-positive/HER2-negative breast cancer--a problem still to be solved. Ann Oncol.

[45] Cardoso F, Van't Veer L, Rutgers E, Loi S, Mook S, Piccart-Gebhart MJ (2008). Clinical application of the 70-gene profile: the MINDACT trial. J Clin Oncol.

[46] Zujewski JA, Kamin L (2008). Trial assessing individualized options for treatment for breast cancer: the TAILORx trial. Future Oncol.

[47] Hall PSSA, Vargas-Palacios A, Stein RC, Bartlett JMS, Marshall A, Rooshenas L, Campbell A, Poole C, Cameron DA, Earl H, Francis A (2014). UK OPTIMA-prelim study demonstrates economic value in more clinical evaluation of multi-parameter prognostic tests in early breast cancer.

[48] Zhao X, Rodland EA, Tibshirani R, Plevritis S (2015). Molecular subtyping for clinically defined breast cancer subgroups. Breast Cancer Res.

[49] Paquet ER, Hallett MT (2015). Absolute assignment of breast cancer intrinsic molecular subtype. J Natl Cancer Inst.

[50] Nielsen T, Wallden B, Schaper C, Ferree S, Liu S, Gao D (2014). Analytical validation of the PAM50-based Prosigna Breast Cancer Prognostic Gene Signature Assay and nCounter Analysis System using formalin-fixed paraffin-embedded breast tumor specimens. BMC Cancer..

[51] Elloumi F, Hu Z, Li Y, Parker JS, Gulley ML, Amos KD (2011). Systematic bias in genomic classification due to contaminating non-neoplastic tissue in breast tumor samples. BMC Med Genomics..

[52] Graham K, Ge X, de Las Morenas A, Tripathi A, Rosenberg CL (2011). Gene expression profiles of estrogen receptor-positive and estrogen receptor-negative breast cancers are detectable in histologically normal breast epithelium. Clin Cancer Res.

[53] Clare SE PI, Mathieson T, Lillemoe HA, Blosser RJ, Choi M, Sauder CAM, Doxey DK, Badve S, Storniolo AMV, Atale R, Radovich M (2012). “Normal” tissue adjacent to breast cancer is not normal. Cancer Res.

[54] Simon RM, Paik S, Hayes DF (2009). Use of archived specimens in evaluation of prognostic and predictive biomarkers. J Natl Cancer Inst.

[55] Sestak I, Dowsett M, Zabaglo L, Lopez-Knowles E, Ferree S, Cowens JW (2013). Factors predicting late recurrence for estrogen receptor-positive breast cancer. J Natl Cancer Inst.

[56] Filipits M, Nielsen TO, Rudas M, Greil R, Stoger H, Jakesz R (2014). The PAM50 risk-of-recurrence score predicts risk for late distant recurrence after endocrine therapy in postmenopausal women with endocrine-responsive early breast cancer. Clin Cancer Res.

