# Supplementary material for: Development and verification of the PAM50-based Prosigna breast cancer gene signature assay
Source: BMC Med Genomics. 2015 Aug 22;8:54. doi: 10.1186/s12920-015-0129-6 (PMC4546262; doi:10.1186/s12920-015-0129-6)
Supplement: Additional file 1: Table S1. — Study inclusion and exclusion criteria for tumor subtyping. (DOCX 30 kb) [file 12920_2015_129_MOESM1_ESM.docx]

**Supplemental Table 1.**  **Study inclusion and exclusion criteria for tumor subtyping**

| Criteria | Metric |
| --- | --- |
| Percent Tumor | FFPE breast cancer tissue sample block with area of highest breast tumor tissue content clearly marked based on H & E stain |
| Tumor Content | Minimum tumor content of 50 % within marked area |
| Number of Tissue Cores | A minimum of two (2) 1.0 mm diameter (with variable depth) punches must be available from within the marked area** |
| Sample ID | Sample identifiable as a unique patient |
| Clinical Data | Clinical data linked to each unique sample (required data is dependent on whether sample will be used for subtype or ROR training) |
| Treatment | Patients have not been treated (i.e. chemotherapeutics) prior to breast tumor excision |
| Sample Selection | Sample is randomly selected for inclusion into this study* |
| *For ROR and risk classification, sample selection was based on a balanced (blinded) subset of 396 of the approximately 1600 BC no AST cohort samples. | |
| ** For the TAM series, RNA was isolated from tissue samples containing a single 1.0 mm diameter punch, but were hybridized only if > 12. 5 ng/uL RNA is obtained per the assay protocol. | |
