# Supplementary material for: Development and verification of the PAM50-based Prosigna breast cancer gene signature assay
Source: BMC Med Genomics. 2015 Aug 22;8:54. doi: 10.1186/s12920-015-0129-6 (PMC4546262; doi:10.1186/s12920-015-0129-6)
Supplement: Additional file 2: Table S2. — Description of FFPE breast cancer patient samples used for training and verifying the Prosigna Algorithm. (DOCX 30 kb) [file 12920_2015_129_MOESM2_ESM.docx]

**Supplemental Table 2.** **Description of FFPE breast cancer patient samples used for training and verifying the Prosigna Algorithm.**

| Sample Cohort Name | Description |
| --- | --- |
| BC no AST | Core punches from areas of FFPE breast tumor blocks identified as viable invasive breast carcinoma containing > 50 % tumor from adjuvant systemic treatment naive patients from the British Columbia Cancer Agency.  Collected for central estrogen receptor (ER) determination during the period from 1986-1992 at the British Columbia provincial referral laboratory, which operated at Vancouver Hospital [18]. British Columbia provincial guidelines from that time period recommended tamoxifen for women > 50 years of age, whose ER status was positive or unknown, and who were either node positive or had lymphovascular invasion (TAM series). Provincial guidelines called for no adjuvant systemic therapy (no AST) for a clinically lower risk group of patients. |
| WashU | Core punches from areas of FFPE breast tumor blocks identified as viable invasive breast carcinoma containing > 50 % tumor during the period from 1997 to 2003 from patients from Washington University and Barnes-Jewish Hospital in St. Louis. |
| UNC | Core punches from areas of FFPE breast tumor blocks identified as viable invasive breast carcinoma containing > 50 % tumor from patients from the University of North Carolina |
| Normal | Scrolls from FFPE breast tissue identified as viable tissue containing 0% tumor obtained from breast reduction mammoplasty patients from the Genetic Pathology Evaluation Centre (University of British Columbia) |
| BC TAM | Core punches from areas of FFPE breast tumor blocks identified as viable invasive breast carcinoma containing > 50 % tumor from Estrogen receptor positive, node negative patients treated with adjuvant tamoxifen from the British Columbia Cancer Agency.  Samples extracted and archived (frozen at -80°C) at Washington University as part of a published study [4] using the BC TAM samples were included in the analysis to increase the overall sample number from the BC TAM study population. |
