# Supplementary material for: Development and verification of the PAM50-based Prosigna breast cancer gene signature assay
Source: BMC Med Genomics. 2015 Aug 22;8:54. doi: 10.1186/s12920-015-0129-6 (PMC4546262; doi:10.1186/s12920-015-0129-6)
Supplement: Additional file 3: Figure S1. — A) Coefficient of variation of each gene, B) interclass correlations for subtype call, and C) replicate ROR-S comparisons. Subtype and ROR-S were generated using the published PAM50 classifier, between NanoString nCounter replicate samples. (PDF 231 kb) [file 12920_2015_129_MOESM3_ESM.pdf]

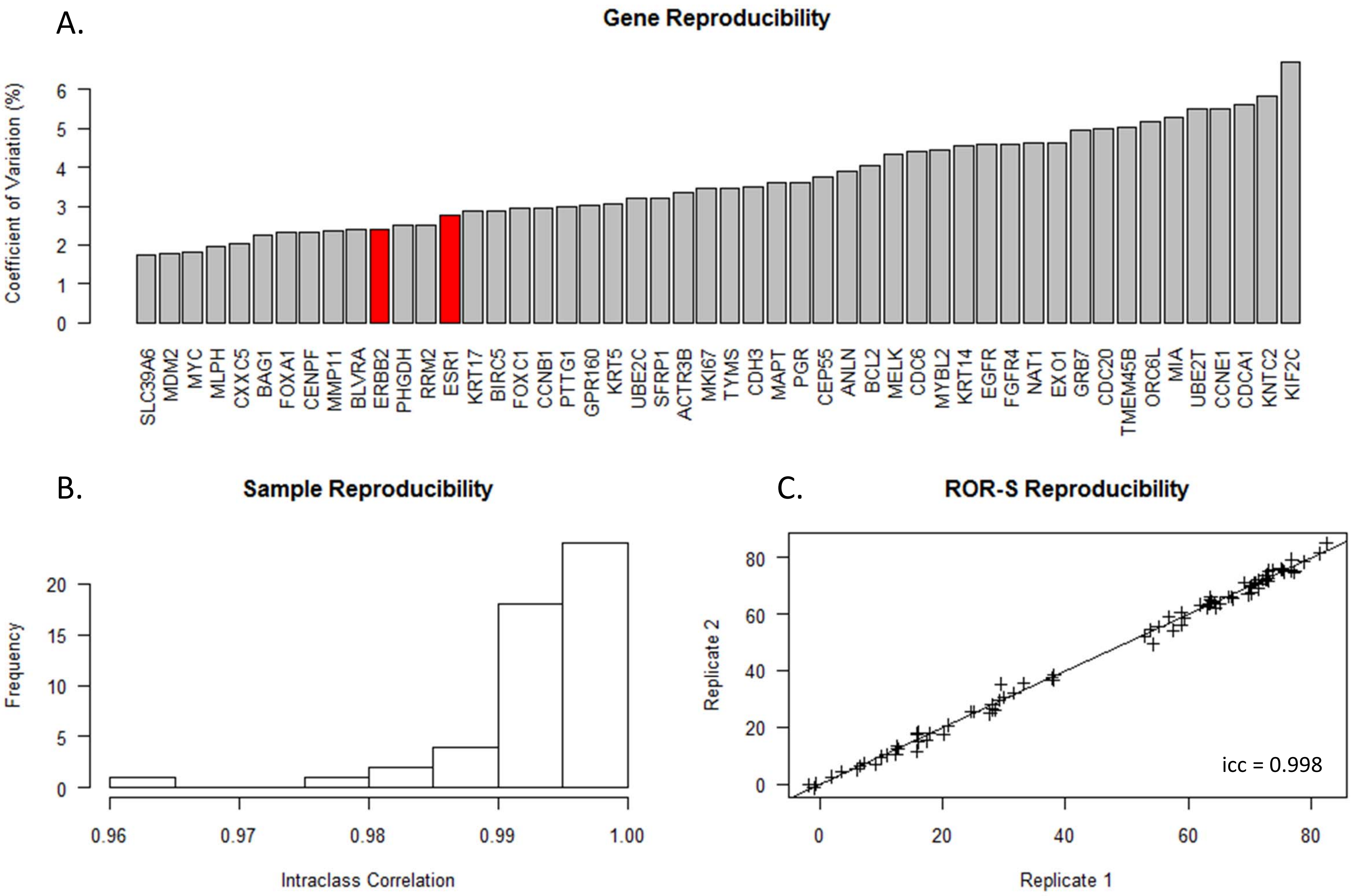
A.
Gene Reproducibility
Coefficient of Variation (%)
0
1
2
3
4
5
6
SLC39A6
MDM2
MYC
MLPH
CXXC5
BAG1
FOXA1
CENPF
MMP11
BLVRA
ERBB2
PHGDH
RRM2
ESR1
KRT17
BIRC5
FOXC1
CCNB1
PTTG1
GPR160
KRT5
UBE2C
SFRP1
ACTR3B
MKI67
TYMS
CDH3
MAPT
PGR
CEP55
ANLN
BCL2
MELK
CDC6
MYBL2
KRT14
EGFR
FGFR4
NAT1
EXO1
GRB7
CDC20
TMEM45B
ORC6L
MIA
UBE2T
CCNE1
CDCA1
KNTC2
KIF2C
B.
Sample Reproducibility
Frequency
0
5
10
15
20
0.96
0.97
0.98
0.99
1.00
Intraclass Correlation
C.
ROR-S Reproducibility
Replicate 2
0
20
40
60
80
Replicate 1
icc = 0.998
