# Supplementary material for: Development and verification of the PAM50-based Prosigna breast cancer gene signature assay
Source: BMC Med Genomics. 2015 Aug 22;8:54. doi: 10.1186/s12920-015-0129-6 (PMC4546262; doi:10.1186/s12920-015-0129-6)
Supplement: Additional file 4: Table S3. — Accuracy estimates (ICC, Slopes, and R-squared) for all genes between nCounter and qRT-PCR. (DOC 57 kb) [file 12920_2015_129_MOESM4_ESM.doc]

**Supplemental Table 3.** Accuracy estimates (ICC, Slopes, and R-squared) for all genes between nCounter and qRT-PCR.

| Gene | R-squared | Slope | ICC |
| --- | --- | --- | --- |
| ACTR3B | 0.828 | 1.114 | 0.901 |
| ANLN | 0.73 | 0.984 | 0.854 |
| BAG1 | 0.317 | 0.299 | 0.394 |
| BCL2 | 0.883 | 0.996 | 0.94 |
| BIRC5 | 0.952 | 1.041 | 0.974 |
| BLVRA | 0.898 | 0.989 | 0.947 |
| CCNB1 | 0.859 | 0.936 | 0.926 |
| CCNE1 | 0.889 | 0.922 | 0.939 |
| CDC20 | 0.829 | 0.934 | 0.907 |
| CDC6 | 0.858 | 0.898 | 0.923 |
| CDCA1 | 0.859 | 0.796 | 0.906 |
| CDH3 | 0.865 | 0.929 | 0.923 |
| CENPF | 0.472 | 0.638 | 0.657 |
| CEP55 | 0.834 | 0.972 | 0.913 |
| CXXC5 | 0.478 | 1.283 | 0.654 |
| EGFR | 0.615 | 0.77 | 0.768 |
| ERBB2 | 0.946 | 1.078 | 0.969 |
| ESR1 | 0.764 | 0.767 | 0.812 |
| EXO1 | 0.459 | 0.318 | 0.47 |
| FGFR4 | 0.492 | 0.627 | 0.641 |
| FOXA1 | 0.88 | 0.821 | 0.916 |
| FOXC1 | 0.75 | 0.957 | 0.844 |
| GPR160 | 0.793 | 0.823 | 0.865 |
| GRB7 | 0.936 | 0.971 | 0.966 |
| KIF2C | 0.715 | 0.846 | 0.836 |
| KNTC2 | 0.837 | 0.978 | 0.915 |
| KRT14 | 0.771 | 0.685 | 0.824 |
| KRT17 | 0.739 | 0.73 | 0.82 |
| KRT5 | 0.613 | 0.647 | 0.715 |
| MAPT | 0.853 | 0.671 | 0.843 |
| MDM2 | 0.686 | 1.007 | 0.829 |
| MELK | 0.889 | 0.867 | 0.934 |
| MIA | 0.403 | 0.427 | 0.546 |
| MKI67 | 0.765 | 0.921 | 0.873 |
| MLPH | 0.781 | 0.867 | 0.874 |
| MMP11 | 0.94 | 0.965 | 0.965 |
| MYBL2 | 0.567 | 0.414 | 0.567 |
| MYC | 0.915 | 1.028 | 0.956 |
| NAT1 | 0.681 | 0.7 | 0.791 |
| ORC6L | 0.735 | 0.514 | 0.703 |
| PGR | 0.728 | 0.623 | 0.763 |
| PHGDH | 0.892 | 0.973 | 0.945 |
| PTTG1 | 0.852 | 0.988 | 0.92 |
| RRM2 | 0.749 | 0.917 | 0.864 |
| SFRP1 | 0.617 | 0.834 | 0.777 |
| SLC39A6 | 0.965 | 1.032 | 0.981 |
| TMEM45B | 0.536 | 0.455 | 0.601 |
| TYMS | 0.94 | 1.002 | 0.968 |
| UBE2C | 0.794 | 1.032 | 0.89 |
| UBE2T | 0.802 | 0.881 | 0.882 |
