# Supplementary figures and images for: Development and verification of the PAM50-based Prosigna breast cancer gene signature assay
Source: BMC Med Genomics. 2015 Aug 22;8:54. doi: 10.1186/s12920-015-0129-6 (PMC4546262; doi:10.1186/s12920-015-0129-6)

**ROR-S**

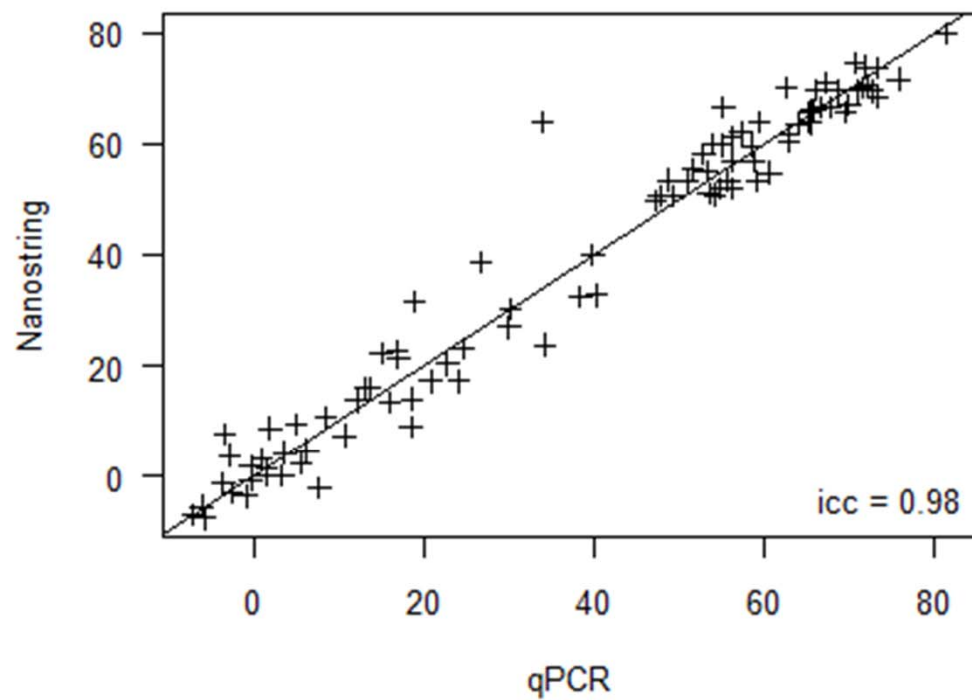

**Proliferation Score**

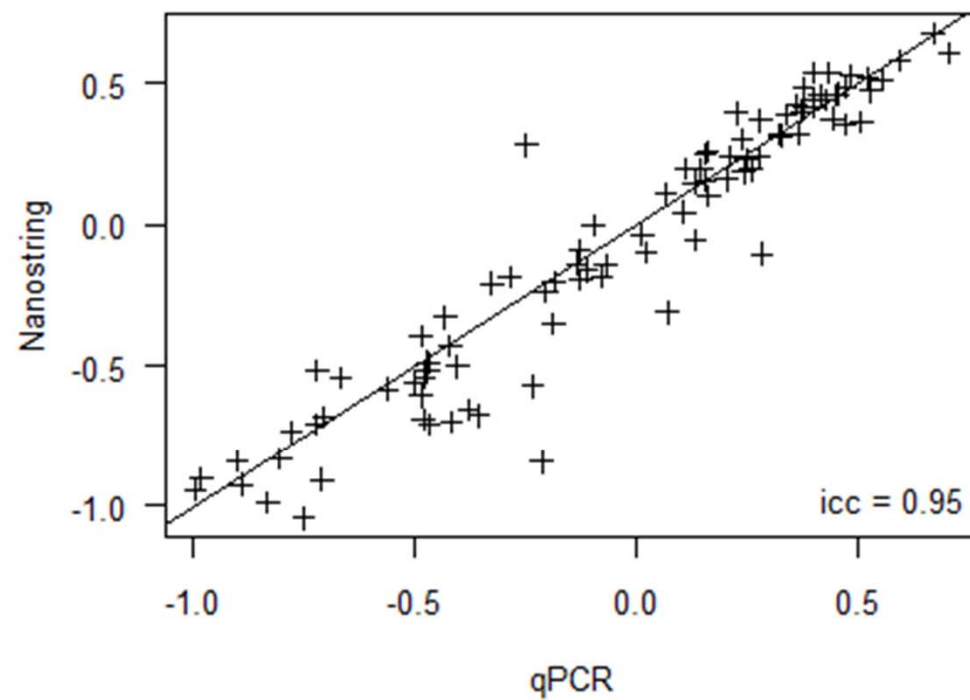

Supplement: Additional file 5: Figure S2. — Comparison of ROR-S and proliferation between NanoString nCounter and PCR data. ROR-S and proliferation scores were generated using the published PCR-based PAM50 classifier. (PDF 101 kb) [file 12920_2015_129_MOESM5_ESM.pdf]

A.

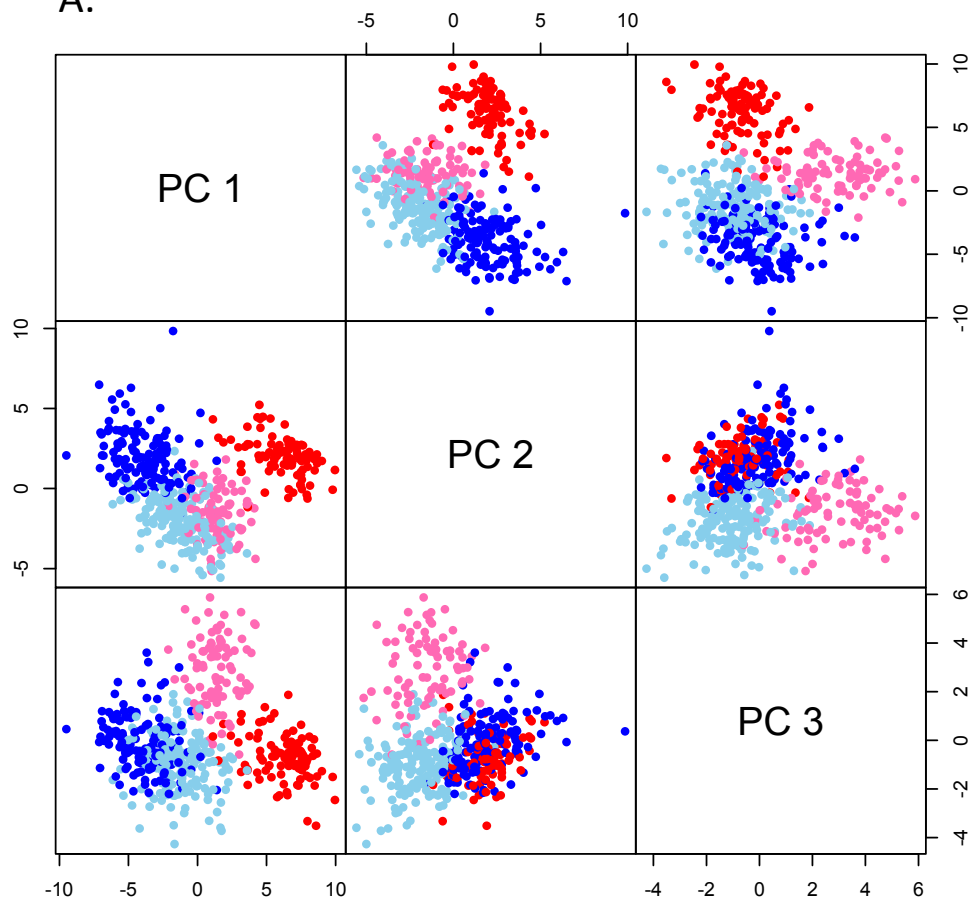

■ Basal-like    ■ HER2-enriched    ■ Luminal A    ■ Luminal B

B.

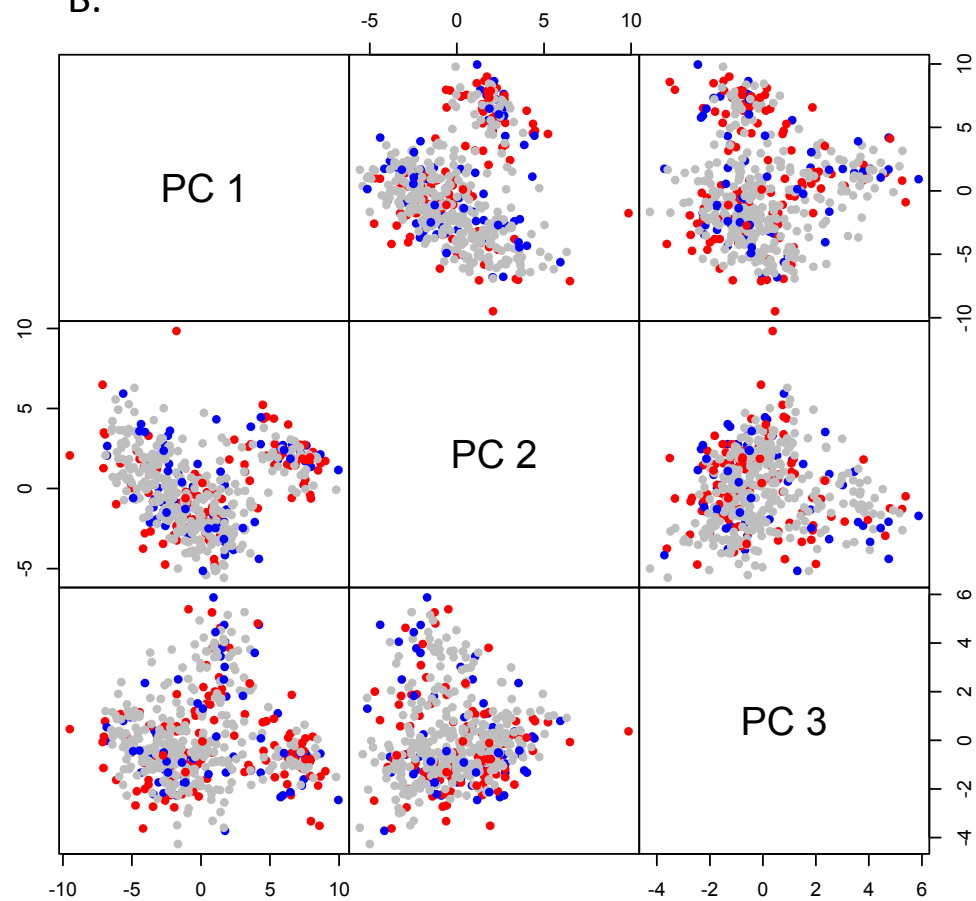

■ UNC    ■ WashU    ■ BC no AST

Supplement: Additional file 7: Figure S3. — Principal component analysis performed on the gene expression data from the training cohorts. Data from each sample are colored by subtype classification (A) or cohort (B). (PDF 114 kb) [file 12920_2015_129_MOESM7_ESM.pdf]

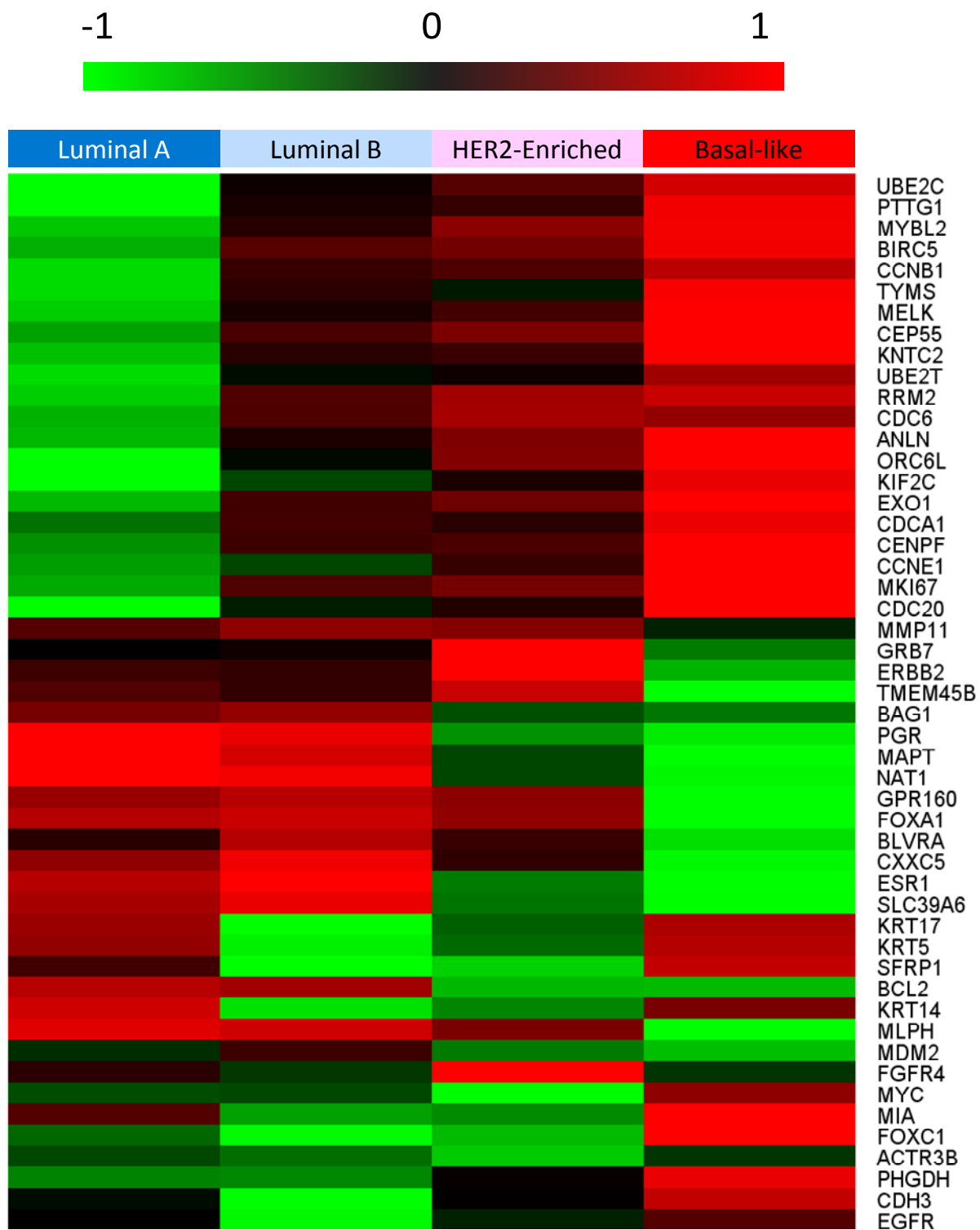

Supplement: Additional file 8: Figure S4. — Four tumor subtype centroids of the Prosigna test. Data are Log2 transformed, reference sample and geomean normalized, and gene scaled nCounter data. (PDF 136 kb) [file 12920_2015_129_MOESM8_ESM.pdf]

## BC TAM, BC No AST, and NKI

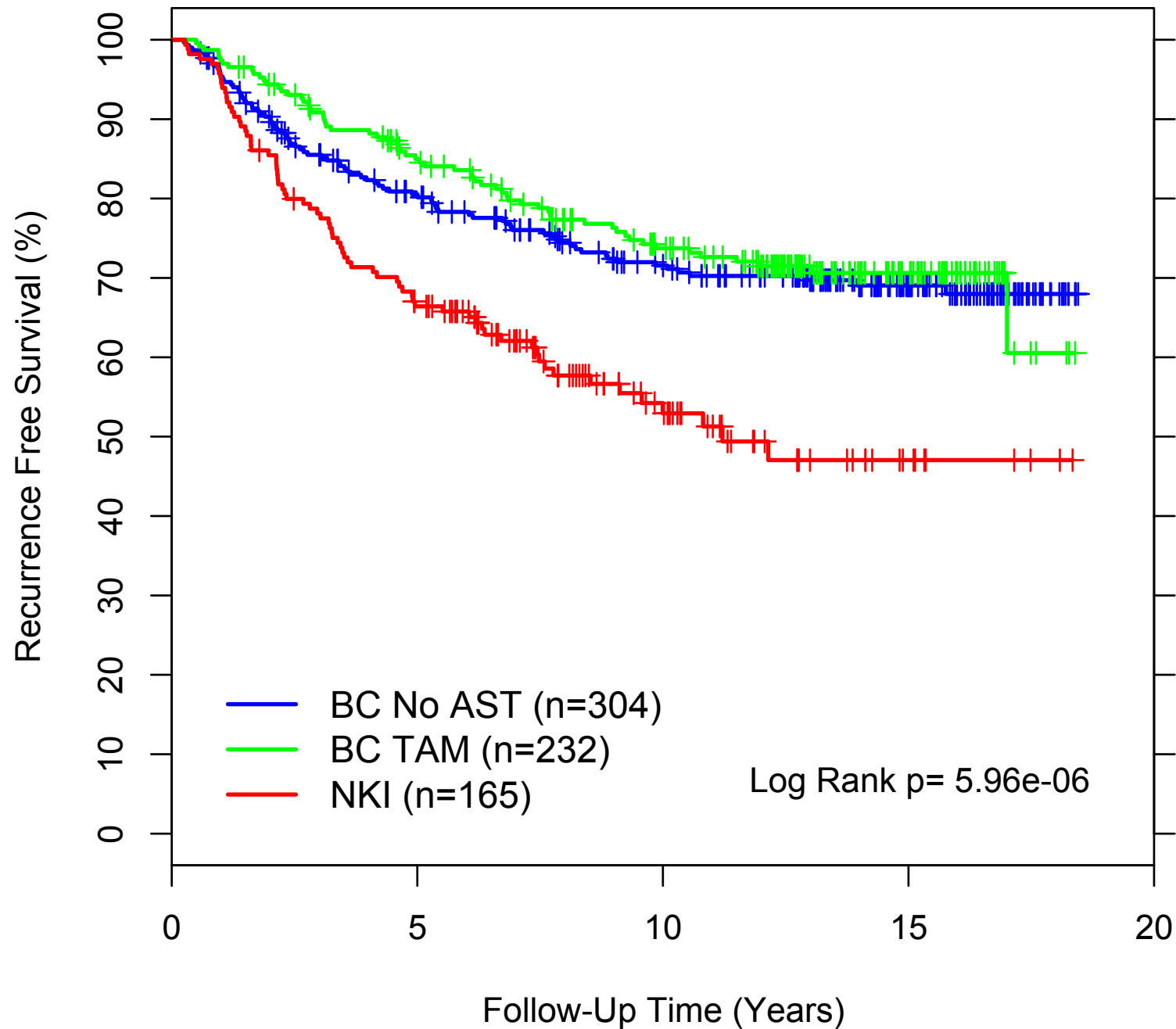

Supplement: Additional file 9: Figure S5. — RFS Kaplan–Meier plot for BC No AST, BC TAM, and NKI cohorts. Cohort colors and numbers of patients are included in the plot along with the results from the Log Rank test. (PDF 59 kb) [file 12920_2015_129_MOESM9_ESM.pdf]

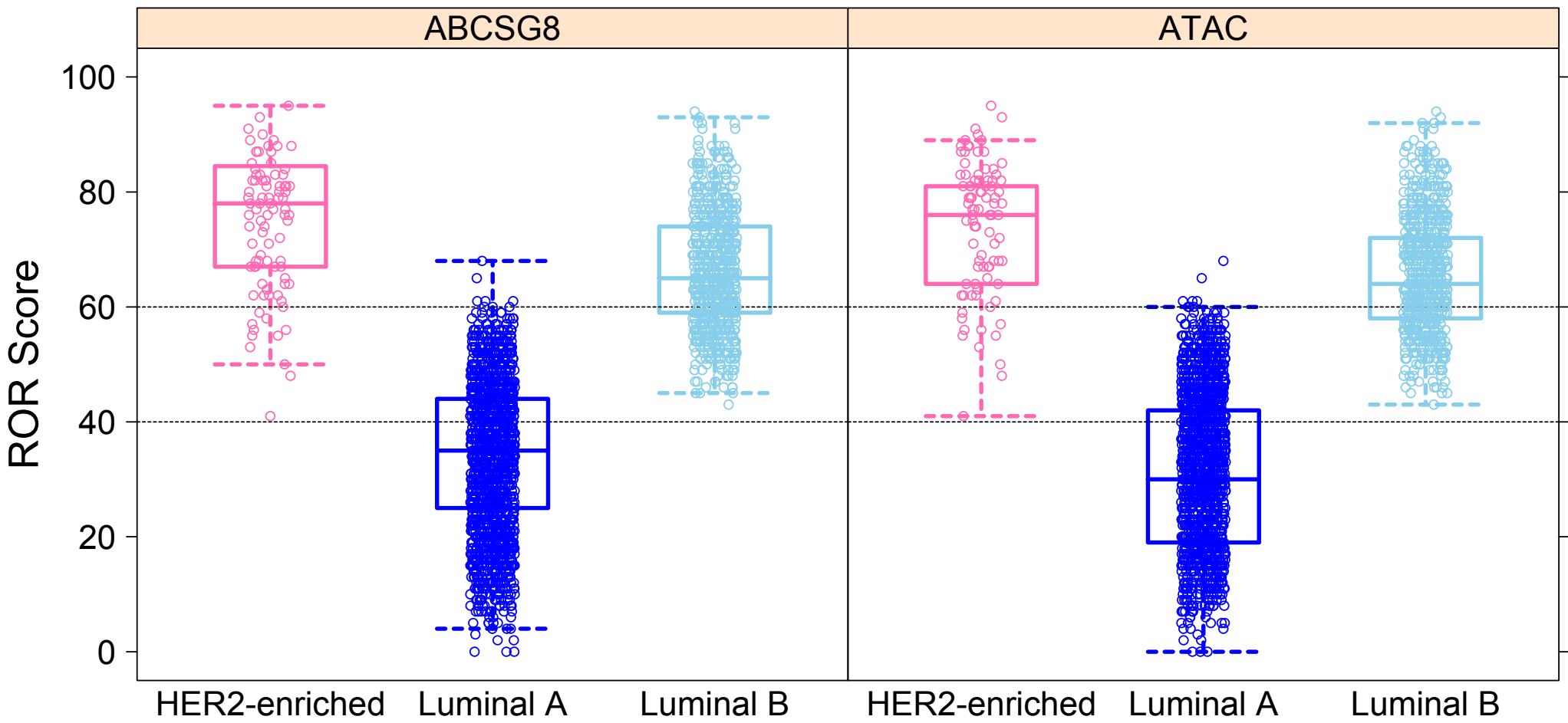

Supplement: Additional file 11: Figure S6. — Distribution of the ROR scores for ABCSG8 and transATAC patient tumor samples. The limits of the boxes represent the first and third quartile and the whiskers represent +/−1.58 IQR/sqrt(n) for each of the three subtypes shown. The horizontal dashed lines illustrate the ROR cutoffs for low/intermediate and intermediate/high risk for N0 patients. Individual points are jittered for illustration purposes. (PDF 83 kb) [file 12920_2015_129_MOESM11_ESM.pdf]
