# Supplementary material for: Development and verification of the PAM50-based Prosigna breast cancer gene signature assay
Source: BMC Med Genomics. 2015 Aug 22;8:54. doi: 10.1186/s12920-015-0129-6 (PMC4546262; doi:10.1186/s12920-015-0129-6)
Supplement: Additional file 6: Table S4. — Number of patient samples from each of the three training cohorts. Numbers represent patient samples used to define each of the four tumor centroids. (DOCX 27 kb) [file 12920_2015_129_MOESM6_ESM.docx]

**Supplemental Table 4. Number of patient samples from each of the three training cohorts.** Numbers represent patient samples used to define each of the four tumor centroids.

| Cohort | Basal-like | HER2-enriched | Luminal A | Luminal B |
| --- | --- | --- | --- | --- |
| UNC | 18 | 16 | 8 | 13 |
| WASHU | 27 | 16 | 4 | 26 |
| BC no AST | 28 | 53 | 38 | 64 |
