# Supplementary material for: Development and verification of the PAM50-based Prosigna breast cancer gene signature assay
Source: BMC Med Genomics. 2015 Aug 22;8:54. doi: 10.1186/s12920-015-0129-6 (PMC4546262; doi:10.1186/s12920-015-0129-6)
Supplement: Additional file 10: Table S5. — Genes used to calculate the Prosigna proliferation score. (DOC 28 kb) [file 12920_2015_129_MOESM10_ESM.doc]

Supplemental Table 5. Genes used to calculate the Prosigna proliferation score.

| *ANLN* | *CEP55* | *ORC6L* |
| --- | --- | --- |
| *CCNE1* | *EXO1* | *PTTG1* |
| *CDC20* | *KIF2C* | *RRM2* |
| *CDC6* | *KNTC2* | *TYMS* |
| *CDCA1* | *MELK* | *UBE2C* |
| *CENPF* | *MKI67* | *UBE2T* |
